# Impact of repeated nasal sampling on detection and quantification of SARS-CoV-2

**DOI:** 10.1038/s41598-021-94547-0

**Published:** 2021-07-21

**Authors:** Joshua M. Levy, Jennifer K. Frediani, Erika A. Tyburski, Anna Wood, Janet Figueroa, Russell R. Kempker, Paulina A. Rebolledo, Mark D. Gonzalez, Julie Sullivan, Miriam B. Vos, Jared O’Neal, Greg S. Martin, Wilbur A. Lam, Jesse J. Waggoner

**Affiliations:** 1 The Atlanta Center for Microsystems-Engineered Point-of-Care Technologies, Atlanta, GA USA; 2 Department of Otolaryngology-Head and Neck Surgery, Emory University School of Medicine, Atlanta, GA USA; 3 Nell Hodgson Woodruff School of Nursing, Emory University, Atlanta, GA USA; 4 Georgia Institute of Technology, Atlanta, GA USA; 5 Department of Medicine, Emory University School of Medicine, Atlanta, GA USA; 6 Rollins School of Public Health, Emory University, Atlanta, GA USA; 7 Department of Pediatrics, Emory University School of Medicine, Atlanta, GA USA; 8 Children’s Healthcare of Atlanta, Atlanta, GA USA; 9 Department of Pathology and Laboratory Medicine, Emory University School of Medicine, Atlanta, GA USA; 10 Aflac Cancer & Blood Disorders Center at Children’s Healthcare of Atlanta, Atlanta, GA USA; 11 Wallace H. Coulter Department of Biomedical Engineering, Emory University and Georgia Institute of Technology, Atlanta, GA USA

**Keywords:** Viral infection, Laboratory techniques and procedures

## Abstract

The impact of repeated sample collection on COVID-19 test performance is unknown. The FDA and CDC currently recommend the primary collection of diagnostic samples to minimize the perceived risk of false-negative findings. We therefore evaluated the association between repeated sample collection and test performance among 325 symptomatic patients undergoing COVID-19 testing in Atlanta, GA. High concordance was found between consecutively collected mid-turbinate samples with both molecular (n = 74, 100% concordance) and antigen-based (n = 147, 97% concordance, kappa = 0.95, CI = 0.88–1.00) diagnostic assays. Repeated sample collection does not decrease COVID-19 test performance, demonstrating that multiple samples can be collected for assay validation and clinical diagnosis.

## Introduction

Diagnostic testing for SARS-CoV-2 is essential for guiding acute-phase clinical management, initiation or continuation of patient isolation, and the need for epidemiologic surveillance and community response. Ensuring accurate test results is of utmost importance to combat the COVID-19 pandemic. Standard of care (SOC) COVID-19 testing in the acute-phase relies on real-time reverse transcription polymerase chain reaction (rRT-PCR) or antigen detection. Per Centers for Disease Control and Prevention (CDC) guidance, such tests can be performed on a variety of samples, including nasopharyngeal (NP), nasal [mid-turbinate (MT) and anterior nares], and oropharyngeal swabs. SARS-CoV-2 detection is highly dependent on the type and quality of specimen obtained^1^. In all cases, specimen collection involves removing potentially limited diagnostic material from the patient^2^. If multiple samples are required for testing, there is concern that viral detection will be significantly reduced in the second sample, leading to false-negative results for certain methods or different pathogens.

The issue of repeat testing comes to the fore in two important scenarios: device evaluation and clinical testing. New SARS-CoV-2 diagnostics under evaluation are frequently designed for near-care, point-of-care, or in-home use. As a result, these come as test kits with engineered swabs and reaction vessels that may not be compatible with routine swabs collected for SOC methods. Because of the possibility of “taking away the virus/sample”, the Federal Drug Administration (FDA) and CDC recommended that SOC NP swabs be collected first during a clinical comparison study between a new device and the SOC. Clinically, the etiology of respiratory tract infections are often indistinguishable, and testing may require multiple tests from separate specimens of the same anatomical site. The FDA/CDC recommendation for the order of sample collection raises the dilemma for clinicians regarding which sample, for which pathogen, to collect first and if diagnostic yield in the second sample will be affected. This will be a consideration whenever multiple swabs are necessary for SOC testing.

The current study sought to address concerns regarding the reproducibility of both SARS-CoV-2 rRT-PCR and antigen testing on paired MT swabs obtained during a single encounter for both clinical care and as a center in the NIH Rapid Acceleration of Diagnostics program tasked with evaluating new rapid tests. We hypothesized that repeated collection of consecutive MT samples does not impact test sensitivity for either amplification or antigen-based assays.

## Results

### Patient population

There were 325 participants. Demographics including participant age, sex, race and number of days since symptom onset are in Table 1.

**Table 1:** Overall, MT rRT-PCR group and MT antigen group participant demographics.

Categories	Overall (N = 325)	MT rRT-PCR (N = 178)	MT antigen (N = 147)
**Total, n (%)**			
Adult	196 (60.3%)	100 (56.2%)	96 (65.3%)
Pediatric	129 (39.7%)	78 (43.8%)	51 (34.7%)
**Age, years**			
Mean (min–max)	33.1 (0.1–82.6)	32.1 (0.1–82.6)	34.3 (0.4–78.3)
Median (25th–75th)	33.6 (9.3–54.8)	28.8 (9.5–55.2)	36.8 (9.0–54.2)
**Gender, n (%)**			
Male	164 (50.5%)	91 (51.1%)	73 (49.7%)
Female	161 (49.5%)	87 (48.9%)	74 (50.3%)
**Race, n (%)**			
White	110 (33.8%)	69 (38.8%)	41 (27.9%)
Black/African American	164 (50.5%)	81 (45.5%)	83 (56.5%)
Asian	17 (5.2%)	12 (6.7%)	5 (3.4%)
Other	34 (10.5%)	16 (9.0%)	18 (12.2%)
**Ethnicity, n (%)**			
Hispanic	44 (13.5%)	24 (13.5%)	20 (13.6%)
Non-hispanic	281 (86.5%)	154 (86.5%)	127 (86.4%)
**Days post-symptom onset**			
Mean (min–max)	3.5 (0.0–7.0)	3.4 (0.0–7.0)	3.6 (0.0–7.0)
Median (25th–75th)	3.0 (2.0–5.0)	3.0 (2.0–5.0)	3.0 (2.0–5.0)
**NP result, n (%)**			
Positive	128 (39.4%)	74 (41.6%)	54 (36.7%)
Negative	197 (60.6%)	104 (58.4%)	93 (63.3%)

*Missing:* n = 1 for race/ethnicity (refused to answer).

### Molecular

**Figure 1:**
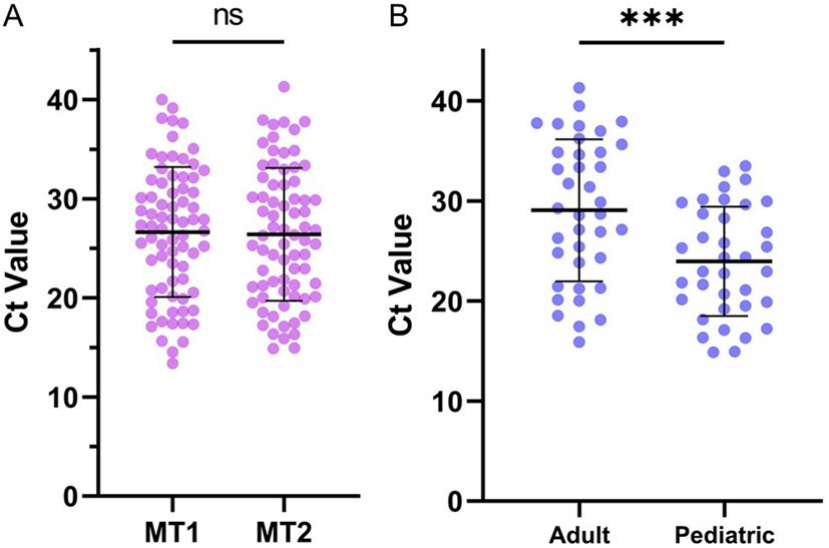
Repeated mid-turbinate sampling does not impact test performance. (**A**) MT1 vs. MT2 mean Ct values did not differ significantly (p = 0.21). (**B**) MT1 Ct values for adult and pediatric participants; pediatric Ct values were significantly lower than adult Ct values (p < 0.01, adjusted for days post-symptom onset).

There was 100% concordance of rRT-PCR results among 74 NP positive patients between MT1 and MT2 tests (72 positive and 2 negative pairs). Additionally, the average Ct value did not differ significantly between MT1 and MT2 samples [median (IQR): MT1 27.09 (20.87–31.30), MT2 26.03 (20.89–31.57), p = 0.21; Fig. 1A]. Ct values among pediatric participants were, on average, lower than Cts for adults even after adjusting for days post-symptom onset [median (IQR): pediatric 22.96 (19.53–29.64), adult 28.69 (23.84–34.84), p < 0.01; Fig. 1B]. This did not impact concordance between MT1 and MT2 rRT-PCR results. 97.3% (72/74) concordance was demonstrated between the results of molecular tests at MT and NP sites.

### Antigen

There was 97% concordance among 147 patients with MT1 and MT2 antigen tests, including 39 positive and 104 negative pairs (95% positive concordance, 98% negative concordance; Table 2). The sequence of results among the four discordant pairs were as follows: two MT1 positive/MT2 negative, one MT1 negative/MT2 positive and one MT1 indeterminate/MT2 positive. Among discordant MT antigen pairs, the mean associated RT-PCR Ct value was 25.61 (range 19.04–31.4). After excluding samples with discordant MT1 and MT2 antigen results (n = 4), 97.3% correlation was found between test results from from MT and NP sources.

**Table 2:** Impact of repeated sample collection on test performance.

	MT2 positive	MT2 negative	MT2 indeterminate	Total
**a. Concordance between paired MT rRT-PCR samples (N = 74)**				
MT1 positive	72	0	0	72
MT1 negative	0	2	0	2
MT1 indeterminate	0	0	0	0
Total	72	2	0	74
**b. Concordance between paired MT antigen samples (N = 147)**				
MT1 positive	39	2	0	41
MT1 negative	1	104	0	105
MT1 indeterminate	1	0	0	1
Total	41	106	0	147

	NP rRT-PCR positive	NP rRT-PCR negative	Total	
**c. Concordance between paired MT antigen samples and NP rRT-PCR (N = 143**^**a**^**)**				
MT1 + MT2 antigen positive	37	2	39	
MT1 + MT2 antigen negative	14	90	104	
Total	51	92	143	

a: McNemar’s test for agreement of positive/negative results: *N/A* No discordant pairs.

a: Kappa coefficient (exact 95% CI): 1.00 (N/A).

a: Overall agreement (exact 95% CI): 100% (N/A).

b: McNemar’s test for agreement of positive/negative results: p = 0.56.

b: Kappa coefficient (exact 95% CI): 0.95 (0.88–1.00).

b: Overall agreement (exact 95% CI): 97% (0.93–0.99).

c: Sensitivity (exact 95% CI): 0.73 (0.58–0.84); Specificity (exact 95% CI): 0.98 (0.92–1.00).

c: McNemar’s test for agreement of positive/negative results: p = 0.003.

c: Kappa coefficient (exact 95% CI): 0.74 (0.63–0.86).

c: Overall agreement (exact 95% CI): 88.8% (0.82–0.93).

^a^Discordant MT1 and MT2 antigen results excluded (n = 4).

## Discussion

Both the FDA and CDC have expressed concern that the sensitivity of SARS-CoV-2 detection may decline in sequentially collected samples. Previous device evaluations employed a number of study designs to address this issue, including the use of different nares^3^, distinct anatomical sites^4,5^, or alternating the order of sample collection^6^. However, in clinical practice, such measures are not possible and may introduce different biases, such as diagnostic yield between different nares or specimen types^7^. The current study demonstrates high concordance between sequential MT samples collected during a single clinical encounter, including complete concordance of results among 74 paired samples tested by rRT-PCR and 97% concordance among paired samples tested for SARS-CoV-2 antigen. These data demonstrate there is ample SARS-CoV-2 virus across a range of viral loads (based on Ct values) to allow for repeated MT sampling if required for multiple tests. In addition, these data support the use of MT sampling as a less invasive collection method for point-of-care and potentially at home testing, with 88.8% concordance (exact CI = 0.82–0.93) between MT antigen test and NP rRT-PCR results.

The current study focused on MT samples, which are being increasingly used for SARS-CoV-2 diagnostic testing^3^. These results cannot necessarily be generalized to the collection of other nasal or oral specimens. Additionally, we did not vary the order of the nasal sampling with SOC NP sampling. In pediatric patients, NP samples were always last and in adults they were always first. Despite these limitations, this study provides proof that SARS-CoV-2 diagnostic test performance is maintained among multiple MT samples when taken at the same encounter. These data have implications for clinical testing algorithms and the design of future device evaluation studies.

## Methods

### Clinical samples

Paired, sequential MT swabs were obtained from 325 participants from COVID-19 testing centers utilized by the Atlanta Center for Microsystems Engineered Point-of-Care Technologies (ACME-POCT) network and affiliated with Emory University, Grady Memorial Hospital and Children’s Healthcare of Atlanta. Inclusion criteria were symptomatic respiratory illness for ≤ 7 days and a documented SARS-CoV-2 molecular test obtained within 24 h of study enrollment. SOC molecular tests differed by site and included the Roche Cobas 6800, Abbott Alinity, and Panther Fusion. NP swabs obtained for SOC testing were collected prior to MT swabs in adults and after in children. Exclusion criteria included inability to tolerate MT swabs or provide informed consent. Clinical and demographic variables were collected in web-based databases (REDCap, Nashville, TN). The study protocol was approved by the Emory University Institutional Review Board, Children’s Healthcare of Atlanta and the Grady Research Oversight Committee. All experiments were performed in accordance with relevant guidelines and regulations. Informed consent was obtained from all participating subjects, including from a parent and/or legal guardian if the patient was under the age of 18 years.

### SARS-CoV-2 testing

Each participant gave two MT samples plus a NP. Of the first 178 participants, 74 tested positive by NP rRT-PCR and had MT pairs tested by rRT-PCR for the current study. MT pairs from the remaining 147 participants were tested for antigen (Supplementary Figure 1). MT swabs for rRT-PCR testing were collected using flocked tapered swabs (Copan FLOQSwab, Ref # 520CS01)^8^. Swabs were placed in 1 mL of saline in a sterile tube. Samples were stored at 4 °C for up to 72 h or − 80 °C if longer delays were expected. Samples were extracted from 500 µL of saline on an eMAG instrument (bioMeriéux) and eluted in 50 µL of buffer. Eluates were tested in an internally controlled, duplex rRT-PCR for the SARS-CoV-2 N2 target and RNase P. Oligonucleotide concentrations, master mix preparation, and cycling conditions were maintained from a previously published, triplex assay that included these two targets^9^. Paired MT swabs were tested side-by-side on a single run.

Sample collection was the same for antigen tests and were completed using the Abbott BinaxNOW™ COVID-19 Ag Card Home Test (Abbott Laboratories, Abbott Park, IL). Results were recorded for both completed tests.

### Statistical analysis

Statistical analyses were performed using SAS 9.4 (Cary, NC). Agreement between the first (MT1) and second MT (MT2) antigen results was assessed using Cohen’s Kappa coefficient with exact 95% confidence intervals. McNemar’s test evaluated overall differences in MT1 vs. MT2 antigen results. Paired t-tests were used for MT1 vs. MT2 Ct comparisons and Wilcoxon signed-rank tests were used for overall adult vs. pediatric Ct comparisons.

## Supplementary Information

Supplementary Figure 1.
