# Supplementary material for: Impact of repeated nasal sampling on detection and quantification of SARS-CoV-2
Source: Sci Rep. 2021 Jul 21;11:14903. doi: 10.1038/s41598-021-94547-0 (PMC8295385; doi:10.1038/s41598-021-94547-0)

Supplemental Figure

***Impact of Repeated Nasal Sampling on Detection and Quantification of SARS-CoV-2***

Supplemental Figure 1. Flowchart of participants


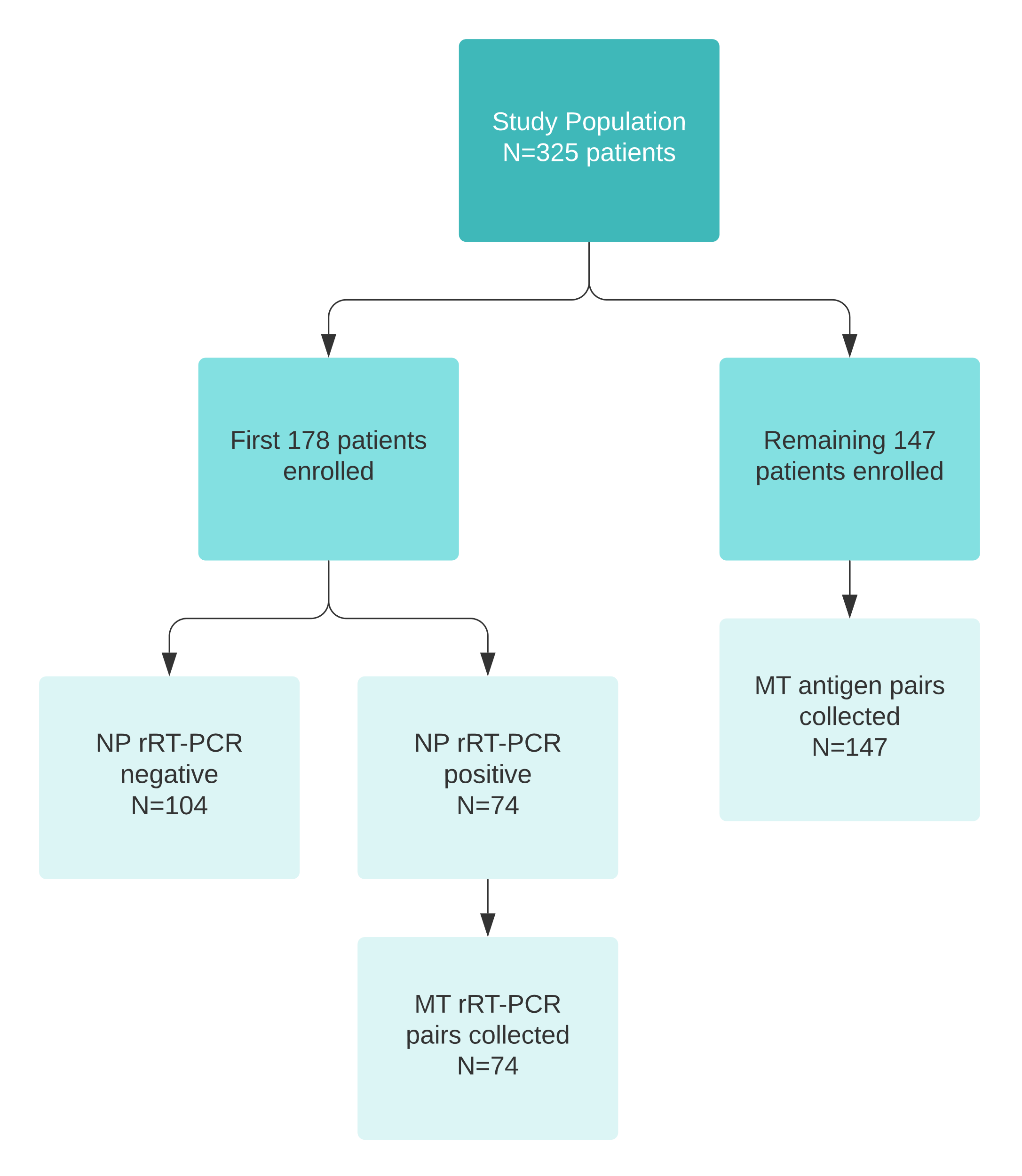

Supplement: Supplementary file 1 — Supplementary Figure 1. [file 41598_2021_94547_MOESM1_ESM.docx]
